# An Observational Study of Human Leptospirosis in Seychelles

**DOI:** 10.4269/ajtmh.19-0228

**Published:** 2020-07-20

**Authors:** Leon Biscornet, Jeanine de Comarmond, Jastin Bibi, Patrick Mavingui, Koussay Dellagi, Pablo Tortosa, Frédéric Pagès

**Affiliations:** 1 Université de La Réunion, UMR PIMIT (Processus Infectieux en Milieu Insulaire Tropical), INSERM U 1187, CNRS 9192, IRD 249, Plateforme de Recherche CYROI, Sainte Clotilde, France;; 2 Infectious Disease Surveillance Unit, Seychelles Public Health Laboratory, Public Health Authority, Ministry of Health, Victoria, Seychelles;; 3 Disease Surveillance and Response Unit, Epidemiology and Statistics Section, Public Health Authority, Ministry of Health, Victoria, Seychelles;; 4 Regional Office of the French Institute for Public Health Surveillance (Santé Publique France), Saint-Denis, France

## Abstract

A 1-year population-based prospective study was launched in Seychelles, a country with one of the highest human incidence of leptospirosis worldwide, to describe the characteristic features of the epidemiology of the disease and highlight the most prominent risk factors. Diagnosis was based on the IgM enzyme-linked immunosorbent assay, microscopic agglutination test, and real-time PCR. A standardized questionnaire was administered to 219 patients aged ≥ 13 years consulting for acute febrile illness. The high incidence of leptospirosis in Seychelles was confirmed. The disease was particularly severe, as the case fatality rate was 11.8%. Leptospirosis was positively associated in univariate analysis with socio-professional and clinical variables including gardening/farming, oliguria, jaundice, conjunctivitis, history of hepatitis C virus infection, anemia, thrombocytopenia, and/or biological renal failure. Epidemiological analyses of the questionnaires highlighted a link of the disease with living in houses (versus apartment), the presence of animals around and in houses, gardening, and misuse of personal protective equipment. Multivariate analyses indicated that being a farmer/landscaper and having cattle and cats around the home are the most significant drivers of leptospirosis. Biological features most associated with leptospirosis were thrombocytopenia, leukocytosis, high values for renal function tests, and elevated total bilirubin. We report changes in behavior and exposure compared with data collected on leptospirosis 25 years ago, with indication that healthcare development has lowered case fatality. Continuous health education campaigns are recommended as well as further studies to clarify the epidemiology of human leptospirosis, especially the role of domestic animals.

## INTRODUCTION

Leptospirosis is an often neglected tropical infectious disease caused by spirochetes of the genus *Leptospira*^1–3^ and is considered as a (re-) emerging zoonosis. Humans are infected when they come in direct or indirect contact with the urine of infected animals.^4,5^ The disease affects more than 1 million persons annually, causing 59,000 deaths^6^ and an estimated 2.9 million disability-adjusted life years lost per annum^7^ reflecting its high socioeconomic impact. Clinical diagnosis of leptospirosis is difficult as symptoms are nonspecific, leading to confusion with other infections such as dengue fever, influenza, and hepatitis, hence contributing to underreporting. Moreover, 90% of cases are asymptomatic or mild.^8^ Symptomatic disease in humans escalates from mild, self-limited febrile illness to severe forms displaying multisystemic complications leading to fulminant life-threatening illness.^9^ Risk factors associated with leptospirosis include behavioral and environmental variables such as rainfall and temperature.^10^ Rodents have traditionally been considered as the main reservoir of *Leptospira* spp., although several other animals (such as cattle, buffaloes, dogs, and cats) can act as reservoirs.^11–14^

Although reported worldwide, leptospirosis is most prevalent in tropical insular countries^15^ where it is of major public health concern, including in the southwestern Indian Ocean islands (SWIOIs) such as Comoros, La Réunion, Mayotte, and Seychelles (where incidence is among the highest worldwide).^16^ High disease incidence in such environments may be due to the warm and humid natural conditions that are conducive to the maintenance and transmission of *Leptospira* spp. In addition, the limited number of animal species typical of insular habitats^17^ may facilitate transmission between competent reservoirs and hence contribute to increase leptospirosis incidence.

Environmental (particularly rainfall and flooding) and behavioral factors are recognized as risk factors for developing leptospirosis. However, the seasonality of the disease in Seychelles has not seen to be as marked as in other locations such as Reunion Island^16,18^ possibly because Seychelles lies closer to the equator.

Molecular investigations have stressed the low diversity of pathogenic *Leptospira* in humans and rats within Seychelles^16^ as both species are infected by *Leptospira interrogans*. Interestingly, despite the high incidence of disease in humans, the *Leptospira* carriage in the rats is low (7.7%). Most importantly, multilocus sequence typing has revealed that sequence types associated with human acute cases or with rat kidney carriage are different and indicate that most (68.7%) of clinical cases have likely not originated from rats.^16^ Last, the highest infection rates in rats are found in nonresidential urban areas. These characteristics highlight that rats are not the main reservoirs of *Leptospira* infecting humans and that an alternative reservoir is yet to be determined in Seychelles.^16^

In the present study, we evaluated the risk factors contributing to leptospirosis in Seychelles and describe the clinical features of the disease and their changing patterns compared with the data reported in previous studies conducted in the country some 25 years ago.

## MATERIALS AND METHODS

### Ethics statement.

The study protocol for humans was reviewed and approved by the Health Research and Ethics Committee of Seychelles (Research Proposal 1405). A written informed consent was provided by all adult patients enrolled in the study or by parents/guardians of minors. All samples were anonymized before laboratory testing.

### Study sites and inclusion criteria.

During December 2014–November 2015, all patients aged ≥ 13 years with febrile illness (≥ 38°C) for more than 3 days at all governmental health facilities (14 clinics, three cottage hospitals, and one referral hospital) in Seychelles were included in an observational study. Patients without fever on the day of inclusion were included if a history of fever in the previous days was documented in their medical file that is, before implementation of antipyretic treatment. The study coordinator center was Seychelles Hospital on Mahé Island. Patients unable to give a good exposure history or to provide clinical information on admission, or refusing blood testing or to participate in the interview were excluded from the study.

### Microbiological investigations and laboratory procedures.

For each included patient, the following biological tests were performed: full blood count, liver function tests, renal function tests, blood culture if hospitalized, and PCR for chikungunya and dengue viruses. Patients who reported a history of travel were also tested for malaria parasites by PCR. For each patient, only maximum values observed during the hospital stay were considered. The biological diagnosis of leptospirosis was performed using real-time (RT) PCR and serological screening through ELISA and the microscopic agglutination test (MAT) following protocols that have been thoroughly described elsewhere.^16^

### Leptospirosis case definition.

A confirmed case of leptospirosis was defined as a suspected case with a positive RT-PCR assay for pathogenic *Leptospira* spp. in blood and/or a positive MAT, a minimum 4 weeks after the onset of symptoms. A positive MAT was defined as one that displayed an infective serogroup with a 4-fold seroconversion in paired sera, or acute sera with a serogroup displaying a minimum titer of 1:400. The presumptive infective serogroup in sera that had co-agglutinating titers was the serogroup displaying two titer orders more than the rest of infecting serogroups.

### Other fever etiologies.

By the end of the study, all clinical records were reviewed to describe the etiology of acute fever in Seychelles. The diagnosis made by the practitioner based on clinical arguments was compared with results of biological investigations. Clinical manifestations and biological disturbances were compared between patients with confirmed leptospirosis and patients with other diseases.

### Clinical and epidemiological investigations.

A questionnaire was administered to eligible outpatients or inpatients included in the study by trained medical personnel (doctors and nurses). This questionnaire included clinical, sociodemographic, current and past medical history, and educational, professional, occupational, environmental, and behavioral variables. Associations between leptospirosis and those variables were analyzed in univariate and multivariate analyses. For alcohol consumption, heavy drinkers were defined as regular drinkers having a calculated average alcohol intake of ≥ 100 mL alcohol per day.^19^

### Statistical analysis.

Data from the administered questionnaire were recorded using EpiData 3.1^®20^ (EpiData Association, Odense, Denmark) and analyzed with R^®^ statistical package^21^ using the chi-squared test or Fisher test for observed frequencies and the *t*-test or Kruskal–Wallis test for continuous data. As leptospirosis cases were compared with a control group of other fevers with regard to the variables of interest, we chose to report odds ratios as a measure of association. Multivariate analysis was performed using a logistic regression model including all variables with a level of significance < 0.20 (*P* < 0.20). A final multivariable logistic regression model was built using a backward stepwise approach. Confounding was assessed when removing variables from the multivariable model. Statistical significance was declared at a likelihood ratio test (*P*-value < 0.05). Plausible two-way interactions between variables retained in the final multivariable model were assessed for significance using a likelihood ratio test. Data of the Population and Housing Census 2010 Report (from the National Bureau of Statistics, Seychelles) and data from the National Survey of Noncommunicable Diseases in Seychelles 2013–2014 (from the Ministry of Health, Seychelles) were used to compare the study sample with the national data.^22,23^

## RESULTS

### Case selection.

During the 12-month study period, 223 febrile patients of 226 eligible were included in the study. Only 219 patients accepted to participate in the study (none of the four patients refusing to participate were positive for *Leptospira*). The epidemiological part of the administered questionnaire could be completed for 209 patients. When leptospirosis cases who died before completion of the form were not included, the nonresponse rates between leptospirosis cases and other causes of fever were not statistically different (6.2% versus 2.4%, Fisher test, *P* = 0.12).

### Review of clinic registers.

Of the 219 patients presenting with acute fever, 197 were males and 22 were females. To understand the reasons of this distorted gender ratio, the clinic registers of attendance of four clinics were reviewed for the study period. These clinics are distributed throughout Mahé Island, which hosts over 90% of the total population: one is located in the north, one in the southeast, one in the west, and one in center of the island. The four selected clinics were representative of other clinics according to their level of attendance and location. Altogether, 75 cases of fever (50 men and 25 women) and 14 suspicions of leptospirosis (10 men and four women) were diagnosed out of 29,391 consultations, representing an incidence of fever of 2.5 per 1,000 consultations and an incidence of suspicion of leptospirosis of 0.5 per 1,000 consultations. In the clinics, male patients accounted for 67% of consultations for fever during the study period. During our study period, there was disproportionate representation of men among the consultants for fever in health centers with a gender ratio of 2.

### Age and gender distribution.

**Figure 1.**
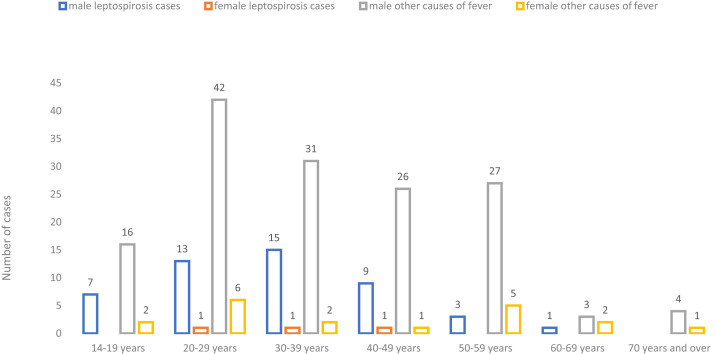
Distribution by age by gender and by leptospirosis infection of the 219 fever cases included, Seychelles 2014–2015. This figure appears in color at www.ajtmh.org.

The mean age of the study sample was 36 years, with no significant difference between genders (40 years for women and 35 years for men, *P* = 0.2, Kruskal–Wallis test). Leptospirosis was diagnosed in 23.3% (51/219) of patients corresponding to an annual incidence of 54.6 (95% CI: 40.7–71.8) per 100,000 population, with 96% (49/51) of cases occurring in men. There was no difference in terms of age between leptospirosis cases and cases because of other causes of fever (33 years [minimum 13; maximum 60] versus 37 years [minimum 13; maximum 80], *P* = 0.3, Kruskal–Wallis test). The distribution by gender, age-group, and leptospirosis infection of the 219 included cases of fever is shown in Figure 1.

### Travel history.

Of the 219 included patients, 31 were not of Seychellois nationality. The positivity rates for leptospirosis were not statistically different (*P* = 0.13, Fisher test) between Seychellois and non-Seychellois patients (25% versus 12.9%). Among the 31 foreigners, four were confirmed leptospirosis cases: three had not recently traveled outside Seychelles, whereas the fourth case occurred in a Malagasy 8 days after his arrival in Seychelles and was probably an imported case from Madagascar.

Travel outside Seychelles was recorded in 213 of 219 patients, with only 10 (4.7%) reporting to have traveled during the study period. Among them, two were further diagnosed as leptospirosis cases: the Malagasy patient returning from Madagascar and a Seychellois returning from Mauritius with a date of onset 16 days after his return. The positivity rates were not significantly different between travelers and those who had not been traveling (20% versus 23.6%, *P* = 0.56, Fisher test).

### Acute fever and leptospirosis incidence by district.

Data by district including the number of included cases, the number of confirmed leptospirosis cases, the inclusion rate, leptospirosis incidence, and percentage of leptospirosis cases are presented in Table 1. Acute fever cases were included from all inhabited districts except La Digue. There was no significant difference in the inclusion rates by district except in La Digue. The incidence of leptospirosis by district was of 54.6 per 100,000 inhabitants (95% CI: 41–72), ranging from 0 in La Digue (95% CI: 0–90) to 136 per 100,000 inhabitants in Anse aux Pins (95% CI: 58–318).

**Table 1:** Number of included cases, number of leptospirosis cases, incidence per 1,000 population of acute fever, and leptospirosis cases by districts in Seychelles, from December 2014 to November 2015.

District	Number of included cases	Number of confirmed leptospirosis cases	District population	Inclusion rate per 1,000 inhabitants	Leptospirosis incidence per 1,000 inhabitants	Percentage of confirmed leptospirosis cases
Anse aux Pins	11	5	3,673	2.99	1.36	45.5
Anse Boileau	12	5	4,183	2.87	1.20	41.7
Anse Etoile	20	3	5,018	3.99	0.60	15.0
Au Cap	6	2	3,743	1.60	0.53	33.3
Anse Royale	13	4	3,818	3.40	1.05	30.8
Baie Lazare	13	3	3,227	4.03	0.93	23.1
Baie st Anne (Praslin)	3	0	3,626	0.83	0.00	0.0
Beau Vallon	12	4	4,142	2.90	0.97	33.3
Bel Air	1	0	3,015	0.33	0.00	0.0
Bel Ombre	3	0	4,163	0.72	0.00	0.0
Cascade	11	4	4,088	2.69	0.98	36.4
Glacis	5	0	4,157	1.20	0.00	0.0
Grand Anse (Mahé)	7	3	2,842	2.46	1.06	42.9
Grand Anse (Praslin)	4	3	4,056	0.99	0.74	75.0
La Digue	0	0	3,506	0.00	0.00	0.0
English River	7	2	4,252	1.65	0.47	28.6
Mont Buxton	5	0	3,173	1.58	0.00	0.0
Mont Fleuri	6	0	3,966	1.51	0.00	0.0
Plaisance	14	2	3,690	3.79	0.54	14.3
Pointe La Rue	11	2	3,245	3.39	0.62	18.2
Port Glaud	7	3	2,378	2.94	1.26	42.9
St. Louis	18	2	3,436	5.24	0.58	11.1
Takamaka	7	0	2,580	2.71	0.00	0.0
Les Mamelles	13	3	2,537	5.12	1.18	23.1
Roche Caiman	9	1	2,893	3.11	0.35	11.1
Total	223	51	93,419	2.39	0.55	22.9

### Occupational categories associated with leptospirosis.

The main work activity was recorded for 213 participants (the distribution of leptospirosis cases by occupation is presented in Table 2). Thirty percent of leptospirosis cases detected in our study were reported in landscapers and farmers, although these work groups accounted for only 12% of our study sample. In these two work groups, almost half of patients consulting for acute fever were ultimately confirmed as leptospirosis cases. When compared with other occupational categories, landscaping and farming were at nearly 5 times the odds of having leptospirosis (OR = 4.9 [95% CI: 2.1–11.5]). When activities classically at higher risk of leptospirosis (landscaping, farming, builders, etc.) are pooled together, they were significantly more represented among the group of leptospirosis cases (61.7%) than in the group of patients with other causes of fever (38.3%): OR = 2.3 [95% CI: 1.2–4.5]. In multivariate analysis, working as a landscaper or farmer was found to be significantly associated with leptospirosis (*P* < 0.0005).

**Table 2:** Number, distribution of leptospirosis cases, and positivity rates by occupation in Seychelles, from December 2014 to November 2015

Occupation	Number of patients included by occupation (percentage of included patients) (*N*, %)	Number of leptospirosis cases by occupation (positivity rates by occupation) (*N*, %)	Distribution of 47 leptospirosis cases by occupation (%)
Farmer or landscaper	26 (12)	14 (54)	30
Builder	32 (15)	3 (9)	6
Mechanic	8 (4)	1 (12)	2
Student	19 (9)	5 (26)	11
Retired	7 (3)	0 (0)	0
Unemployed	15 (7)	3 (20)	6
Other occupations at risk	29 (14)	9 (31)	19
Not at-risk occupations	77 (36)	12 (15)	26

### Hospitalization rate of leptospirosis cases.

Of the 219 patients with acute fever, 173 (79%) were hospitalized and 46 (21%) were treated as outpatients. The hospitalization rate and the mean duration of stay were higher for leptospirosis cases than other causes of fever, respectively, 90% versus 75.6% (OR = 2.9 [95% CI: 1.1–7.9]) and 3.6 days versus 2.9 days (*P* < 0.01). The delay before hospitalization for leptospirosis cases was on average 3.6 days after the onset of symptoms versus 2.6 days for the other causes of fever (*P* < 0.01). In the same way, the delay before the first blood sampling was higher for hospitalized leptospirosis cases: 4.2 days compared with 3 days for the other causes of fever (*P* < 0.01).

### Effect of other disease conditions.

We screened for a possible influence of other diseases or habits on the leptospirosis rate. There was no difference in the clinical history between our sample and the general population of Seychelles except for high blood pressure prevalence and heavy drinking that were significantly lower in our study sample: 10.8% versus 19.3% (*P* < 0.01) and 0.9% versus 11% (*P* < 0.0001), respectively. There was no difference of prevalence between leptospirosis and other causes of fever for most of the listed medical antecedents: diabetes (5.1% of participants), high blood pressure (10.8% of participants), previous history of leptospirosis (2.4% of participants), hepatic diseases (4.2% of participants), renal diseases (0.9% of participants), HIV infection (0.9% of participants), alcoholism (0.9% of participants), intravenous drug use (IVDU; 3.2% of participants), and cardiac diseases (1.4% of participants). Of note, the record of a previous infection by HCV was significantly more frequent in leptospirosis cases (6.2% versus 0.6%; OR = 10.4 [95% CI: 1.1–102.6]).

### Clinical and biological features of leptospirosis.

Clinical and biological features by leptospirosis infection or other causes of fever are presented in Table 3. In univariate analysis, only conjunctivitis, jaundice, oliguria, myalgia–arthralgia, and fever were significantly associated with leptospirosis. In the same way, only cough was clearly infrequent in leptospirosis cases.

**Table 3:** Clinical and biological features by leptospirosis infection or other causes of fever in Seychelles, from December 2014 to November 2015

	Leptospirosis cases	Other causes of fever	P-value	Odds ratio [95% CI]	Adjusted odds ratio* [95% CI]
Clinical features					
Fever confirmed at enrollment	38 (75%)	97 (57%)	*P* < 0.05	2.1 [1.1–4.3]	2.4 [0.9–6.3]
Myalgia–arthralgia	37 (66%)	76 (45%)	*P* < 0.01	3.2 [1.6–6.2]	1.4 [0.5–3.4]
Oliguria	12 (24%)	13 (9%)	*P* < 0.01	3.7 [1.6–8.3]	1.3 [0.4–3.8]
Jaundice	11 (22%)	13 (8%)	*P* < 0.01	3.3 [1.4–7.8]	1.8 [0.6–5.1]
Conjunctivitis	9 (18%)	10 (6%)	*P* < 0.01	3.4 [1.3–8.5]	3.0 [0.9–9.5]
Back pain	17 (34%)	36 (21%)	*P* = 0.08	1.8 [0.9–3.6]	
Hematemesis	3 (6%)	4 (2%)	*P* = 0.21	2.6 [0.5–11.8]	
Melena	0 (0%)	1 (0.6%)	*P* = 0.58	3.3 [0.2–54.4]	
Hemoptysis	3 (6%)	9 (5%)	*P* = 0.88	1.1 [0.3–4.2]	
Hematuria	3 (6%)	6 (4%)	*P* = 0.46	1.7 [0.4–7.0]	
Abdominal pain	10 (20%)	38 (23%)	*P* = 0.64	0.8 [0.4–1.8]	
Headache	21 (42%)	89 (53%)	*P* = 0.14	0.6 [0.3–1.2]	
Meningitis	2 (4%)	6 (4%)	*P* = 0.90	1.1 [0.2–5.6]	
Diarrhea	3 (6%)	9 (5%)	*P* = 0.88	1.1 [0.3–4.2]	
Dyspnea	1 (2%)	2 (1%)	*P* = 0.67	1.6 [0.1–18.7]	
Cough	2 (4%)	24 (14%)	*P* < 0.05	0.2 [0.1–0.9]	0.0 [0.0–1,000]
Biological anomalies†					
Anemia (Hb < 12.5g/dL for women and < 13.5/g/dL for men)	48 (96%)	108 (64%)	*P* < 0.0001	8.9 [2.6–29.7]	0.8 [0.2–4.1]
Severe anemia (Hb < 11g/dL)	19 (38%)	24 (14%)	*P* < 0.001	3.6 [1.7–7.2]	
Thrombocytopenia (< 150,000/µL)	44 (87%)	63 (38%)	*P* < 0.0001	10.4 [4.4–24.6]	19.5 [4.1–91.8]
Leukocytosis (> 10,000/mm3)	34 (66%)	66 (39%)	*P* < 0.001	3.1 [1.6–5.9]	6.0 [1.7–21.4]
Neutrophilia (> 7,000/mm^3^)	44 (87%)	106 (63%)	*P* < 0.001	3.6 [1.6–8.7]	
High urea (> 7 mmol/L)	22 (43%)	10 (6%)	*P* < 0.0001	11.9 [5.1–27.9]	
High creatinine (> 110 µmol/L)	21 (40%)	22 (13%)	*P* < 0.0001	4.6 [2.3–9.5]	
Biological renal failure (glomerular filtration rate < 90 mL/min/1.73 m^2^)	30 (59%)	52 (31%)	*P* < 0.001	3.2 [1.7–6.0]	3.7 [0.7–18.1]
High alkaline phosphatase (> 275 U/L)	21 (40%)	17 (10%)	*P* < 0.0001	6.2 [2.9–13.,1]	
High total bilirubin (> 20 µmol/L)	36 (72%)	53 (31%)	*P* < 0.0001	5.2 [2.7–10.3]	6.7 [1.6–27.8]

* Sex and age have been included in the models and were not significant.

† Different multivariable models have been tested using interactions between variables (i.e., high urea, high creatinine, and biological renal failure) or using one variable by organ (i.e., high urea or high creatinine or biological renal failure). The final model used only one variable by organ. The use of another variable by organ or interactions did not modify the retained variables in models.

Results of biological investigations were available only for 156 patients and were missing for 27 inpatients (15%) and for 36 outpatients (78%). In univariate analysis, anemia, severe anemia (< 11 g/dL), thrombocytopenia, leukocytosis, neutrophilia, urea elevation, creatinine elevation, biological renal failure, alkaline phosphatase elevation, and bilirubin elevation were significantly linked with leptospirosis infection. In multivariate analysis, thrombocytopenia (Adjusted odds ratio [AOR] = 19.5 [95% CI: 4.1–91.8]), leukocytosis (AOR = 6.0 [95% CI: 1.7–21.4]), and elevated total bilirubin (AOR = 6.7 [95% CI: 1.6–27.8]) were still significantly associated with leptospirosis infection.

### Severe and fatal leptospirosis cases.

Death occurred in 11.2% (6/51) of leptospirosis cases versus 1.8% (3/165) in non-leptospirosis cases (OR = 7.2 [95% CI: 1.7–29.9]). Leptospirosis cases were classified into mild or severe forms according to clinical and biological manifestations. Severe forms (21) represented 41.1% of leptospirosis cases: five acute renal failure, four acute renal failure with hepatic failure, four pulmonary hemorrhage, three acute hepatic failure, three acute renal failure with pulmonary hemorrhage, one endocarditis, and one death at arrival.

There was no difference of prevalence between mild and severe leptospirosis forms for all the listed medical backgrounds: diabetes, high blood pressure, leptospirosis, hepatic diseases, renal diseases, HIV infection, HCV infection, alcoholism, IVDU, and cardiac diseases. Among the 16 listed symptoms (fever, back pain, hematemesis, melena, hemoptysis, myalgia–arthralgia, hematuria, oliguria, abdominal pain, jaundice, headache, conjunctivitis, meningitis, cough, diarrhea, and dyspnea), two were significantly more associated with severity: oliguria (40% versus 13.3%; OR = 4.0 [95% CI: 1.1–15.8]) and jaundice (45% versus 6.7%; OR = 10.5 [95% CI: 1.9–56.0]). The case fatality rate (CFR) was not significantly different according to the clinical presentations (9.5% for severe forms versus 6.7% for noncomplicated forms). Some biological anomalies were significantly associated with severe forms: severe anemia (< 11) (70% versus 14.8%; OR = 16.2 [95% CI: 3.9–66.9]), urea elevation (85% versus 11.5%; OR = 54.0 [95% CI: 9.9–297.9]), creatinine elevation (70% versus 18.5%; OR = 12.5 [95% CI: 3.2–48.1]), biological renal failure (85% versus 40.7%; OR = 9.0 [95% CI: 2.2–37.4]), and bilirubin elevation (95% versus 53.9%; OR = 17.5 [95% CI: 2.1–147.6]). The prevalence of thrombocytopenia was not significantly different between simple and severe forms of leptospirosis (80.8% versus 95%), but the severity of thrombocytopenia was significantly higher for severe forms (mean number of platelets 53,000 versus 99,500, *P* = 0.003, Kruskal–Wallis test). There was no difference of prevalence between recovered and deceased leptospirosis cases for all listed medical histories: diabetes, high blood pressure, leptospirosis, hepatic diseases, renal diseases, HIV infection, HCV infection, alcoholism, IVDU, and cardiac diseases. Among the 16 listed symptoms (mentioned previously), only abdominal pain was significantly associated with fatal outcomes: 60% versus 15%; OR = 10.8 [95% CI: 1.6–71.1]). The CFR was not significantly higher in pulmonary leptospirosis than in other forms (33% versus 11%). Severe anemia was more frequent in fatal issue with an average hemoglobin level reaching 8.4 g/dL versus 11.4 g/dL in survivors. The thrombocytopenia was in average more profound in fatal issues (49,000 platelets/µL versus 96,000/µL in survivors), but this difference was not statistically significant.

### Accuracy of clinical diagnoses.

Clinicians established for 121 patients a tentative diagnosis of leptospirosis on clinical grounds. The accuracy of this diagnosis was challenged by results of the biological tests (available for 113 patients). Tentative diagnosis was not retrieved for only three confirmed leptospirosis cases. On the one hand, 38% of patients clinically considered as leptospirosis cases (including mild and severe forms) were not confirmed by the specific tests. On the other hand, 8.7% of confirmed leptospirosis cases were diagnosed by clinicians as endocarditis, cholecystitis, cellulitis, acute gastroenteritis, viral fever, or heroin overdose. The positive predictive value (PPV) of the diagnosis of leptospirosis in the clinical file was only of 62%, and the negative predictive value of other diagnostics was of 90.8%. Among the 65 patients for whom the cause of acute fever was not leptospirosis, infectious diseases accounted for 90.8% (upper respiratory tract infection [URTI], lower respiratory tract infection [LRTI], gastroenteritis, cellulitis, hepatitis, pyelonephritis, dengue, and malaria) and noninfectious diseases represented 9.2% (pancreatitis, urinary lithiasis, polyarthritis, bowel obstruction, gastrointestinal bleeding, and malignant hyperthermia due to exercise).

### Knowledge on leptospirosis.

The level of knowledge of the sample is presented in Table 4 by leptospirosis status. There was globally no difference between groups either in the level of knowledge of the participants or on the mode of contamination of leptospirosis as well as on protective measures. The level of knowledge on leptospirosis was very low in all patients. Similarly, preventive measures were overall poorly known. Knowledge that leptospirosis is a deadly disease was more frequent among cases versus non-cases (92% versus 68%, OR = 4.8 [95% CI: 1.7–14.4]), whereas the absence of leptospirosis prevention knowledge was more common in non-cases than cases (34% versus 16%; OR = 0.3 [95% CI: 0.1–0.8]).

**Table 4:** Level of knowledge on leptospirosis of participants according to their leptospirosis status in Seychelles, from December 2014 to November 2015

	Leptospirosis cases	Other causes of fever	*P*-value	Odds ratio [95% CI]
Global knowledges on leptospirosis				
Has never heard about leptospirosis	12 (27%)	52 (31%)	*P* = 0.51	0.8 [0.4–1.6]
Know that leptospirosis is a deadly disease	41 (92%)	111 (68%)	*P* < 0.001	**4.8 [1.7-14.4]**
Know that leptospirosis is a curable disease	24 (54%)	97 (59%)	*P* = 0.48	0.8 [0.4–1.5]
Knowledge on leptospirosis transmission				
No knowledge on leptospirosis transmission	13 (29%)	56 (34%)	*P* = 0.50	0.8 [0.4–1.6]
Know that walking barefoot is a risk factor for leptospirosis	7 (15%)	31 (19%)	*P* = 0.60	0.8 [0.3–1.9]
Know that contact with garbage is a risk factor for leptospirosis	1 (2%)	8 (5%)	*P* = 0.43	0.4 [0.05–3.6]
Know that rats are involved in leptospirosis transmission	25 (57%)	80 (49%)	*P* = 0.42	1.3 [0.7–2.5]
Know that contamination can be occurred in fresh water	8 (18%)	29 (18%)	*P* = 0.98	1.0 [0.4–2.4]
Knowledge on leptospirosis prevention				
No knowledge on leptospirosis prevention	7 (16%)	56 (34%)	*P* < 0.01	**0.3 [0.1–0.8]**
Avoiding unprotected contact with garbage	1 (2%)	7 (4%)	*P* = 0.52	0.5 [0.06–4.2]
Avoiding unprotected contact with freshwater	2 (4%)	13 (8%)	*P* = 0.42	0.5 [0.1–2.5]
Avoiding garbage accumulation	2 (4%)	11 (7%)	*P* = 0.57	0.6 [0.1–3.0]
Using personal protective equipment	20 (44%)	65 (40%)	*P* = 0.56	0.9 [0.5–1.6]
Wearing shoes outside	13 (28.9%)	48 (29.3%)	*P* = 0.96	0.9 [0.5–2.0]

Statistically significant odds ratios are bolded.

Housing conditions, environmental factors, and behaviors by leptospirosis status are shown in Table 5. Leptospirosis cases were more frequently living in houses than in apartments (87% versus 69%; OR = 3.6 [95% CI: 1.3–9.7]). There was no difference in the frequency of garbage collection, the type of sewage system, the type of soiled water disposal system, the type of waste disposal, the type of houses (type of floor, type of ground, and type of materials used for walls and roofs), the type of water used for drinking or cooking (treated water for 87%), or the type of water used for bathing (treated water for 84%) between leptospirosis and non-leptospirosis cases. The use of personal protective equipment (PPE) during risky activities was very low in our sample both in leptospirosis and non-leptospirosis cases. Among patients using PPE (gloves and boots), those using boots only were more frequent in the leptospirosis group (65% versus 40%; OR = 2.7 [95% CI: 1.3–5.3]), but we did not succeed in identifying a link between PPE use or misuse during risky activities in the 4 weeks preceding the onset of symptoms. In univariate analysis, the presence of animals in the vicinity (especially cattle, cats, poultry, and dogs but not rats) and the presence of pets at home were significantly more frequent around leptospirosis cases. Importantly, with the exception of regular gardening, none of the behaviors classically described as associated with leptospirosis risk in Seychelles or in other places were retrieved in our study. Last, multivariate analysis highlighted the presence of cattle (AOR = 11.7 [95% CI: 1.1–131.4]), the presence of cats (AOR = 4.1 [95% CI: 1.5–10.6]) around homes, and working as a landscaper or farmer (AOR = 5.2 [95% CI: 1.7–16.0]) as significantly associated with leptospirosis cases.

**Table 5:** Housing conditions, environmental factors, and at-risk behaviors by leptospirosis status in Seychelles, from December 2014 to November 2015

	Leptospirosis cases	Other causes of fever	*P*-value	Odds ratio [95% CI]	Adjusted odds ratio [95% CI]
Housing					
Living in a house	40 (87%)	113 (69%)	*P* < 0.01	3.6 [1.3–9.7]	1.8 [0.6–5.7]
Wood house with corrugated sheet	5 (11%)	26 (16%)	*P* = 0.42	0.7 [0.2–1.8]	–
Separate kitchen	4 (9%)	25 (15%)	*P* = 0.27	0.5 [0.2–1.6]	–
Connection to municipal sewerage	1 (2%)	18 (11%)	*P* = 0.07	0.2 [0.02–1.4]	–
Closed bins available	12 (26%)	35 (22%)	*P* = 0.44	1.3 [0.6–2.9]	–
Weekly garbage collection only	5 (11%)	21 (13%)	*P* = 0.76	0.8 [0.3–2.4]	–
Using untreated water	7 (17%)	12 (8%)	*P* = 0.08	2.3 [0.9–6.3]	–
Environmental factors					
Rats in the vicinity	24 (55%)	79 (48%)	*P* = 0.53	1.2 [0.6–2.4]	–
Animals in the vicinity	30 (86%)	109 (67%)	*P* = 0.97	1.0 [0.5–2.0]	–
Dogs in the vicinity	45 (100%)	103 (63%)	*P* < 0.0001	26.6 [3.6–198.3]	0.9 [0.3–3.0]
Cats in the vicinity	45 (100%)	52 (32%)	*P* < 0.0001	96.9 [13.0–722.5]	4.1 [1.5–10.6]
Poultry in the vicinity	30 (67%)	20 (12%)	*P* < 0.0001	14.4 [6.6–31.3]	0.7 [0.2–2.5]
Cattle in the vicinity	30 (67%)	1 (0.7%)	*P* < 0.0001	326 [41.5–2,561.3]	11.7 [1.1–131.4]
Direct interactions with animals	20 (44%)	66 (40%)	*P* = 0.61	1.2 [0.6–2.3]	
Pets at home	17 (38%)	37 (23%)	*P* < 0.05	2.1 [1.0–4.2]	1.6 [0.6–3.9]
Animal bites	1 (2%)	5 (3%)	*P* = 0.76	0.7 [0.5–6.6]	–
River in the vicinity	16 (35%)	52 (32%)	*P* = 0.62	1.2 [0.6–2.4]	–
Previous leptospirosis case at home	4 (9%)	8 (5%)	*P* = 0.30	1.9 [0.5–6.6]	–
Previous leptospirosis case in the vicinity	4 (9%)	7 (4%)	*P* = 0.21	2.2 [0.6–7.8]	–
Behaviors					
Walking barefoot at home	23 (52%)	93 (57%)	*P* = 0.50	0.8 [0.4–1.5]	–
Walking barefoot outside	11 (25%)	36 (22%)	*P* = 0.72	1.1 [0.5–2.5]	–
Swimming in the sea	9 (21%)	28 (17%)	*P* = 0.64	1.2 [0.5–2.8]	–
Swimming in fresh water	5 (11%)	17 (10%)	*P* = 0.88	1.1 [0.4–3.1]	–
Swimming in swimming pools	1 (2%)	1 (0.7%)	*P* = 0.32	3.7 [0.2–60.4]	–
Using river water for bathing	9 (21%)	17 (10%)	*P* = 0.08	2.2 [0.9–5.2]	–
Washing clothes in river	4 (9%)	11 (7%)	*P* = 0.61	1.3 [0.4–4.5]	–
Hiking in swamps	2 (5%)	4 (3%)	*P* = 0.47	1.9 [0.3–10.5]	–
Hiking in forest	6 (14%)	15 (10%)	*P* = 0.40	1.5 [0.5–4.2]	–
Not always washing fruits or vegetables	8 (18%)	44 (27%)	*P* = 0.21	0.6 [0.2–1.4]	–
Regular alcohol consumption	10 (22%)	36 (22%)	*P* = 0.96	1.0 [0.4–2.2]	–
Regular gardening	22 (50%)	48 (30%)	*P* < 0.01	2.3 [1.2–4.5]	1.5 [0.6–4.0]
Working outside the previous 4 weeks	16 (35%)	42 (26%)	*P* = 0.18	1.6 [0.8–3.2]	–
Construction work in the previous 4 weeks	5 (11%)	36 (22%)	*P* = 0.10	0.4 [0.2–1.2]	–
Landscaping or farming	14 (54%)	12 (7%)	*P* < 0.0001	4.9 [2.1–11.5]	5.2 [1.7–16.0]
Presence of wounds	18 (40%)	59 (36%)	*P* = 0.62	1.2 [0.6–2.3]	–
Using boots without gloves as protective equipment	29 (65%)	66 (40%)	*P* < 0.01	2.7 [1.3–5.3]	1.7 [0.7–3.8]

## DISCUSSION

Leptospirosis data used in this study combined RT-PCR, and IgM and MAT screening.^16^ The turnaround time before molecular test results were available was less than 5 days for 75% of non-leptospirosis cases and more than 7 days for only seven suspected cases. The probability for false negatives was hence low and controlled by the additional diagnosis of IgM and MAT, which are sensitive in the second phase of the disease and hence complementary to molecular screening. Therefore, case classification bias in this study, if existing, is probably low. Most cases were autochthonous; hence, the epidemiological patterns highlighted herein are mostly relevant for describing the situation prevailing in Seychelles. As the inclusion rate was the same in all health districts except La Digue, we can consider that the protocol of inclusion was homogenously applied in all districts.

There was an overrepresentation of male patients (96%) in our study sample despite the generic criterion of inclusion (acute fever) which was rather because of a gender bias in the attendance of health facilities than to a bias in patient inclusion. It is well known that access to care is sometimes more difficult for women in low- to middle-income countries, including admission to the ICU or hospitalization.^24,25^ The reasons for this difference are still not clearly understood and include difference in clinical presentations or decision-making. This bias is actually quite similar to that reported in 1992 (89%)^26^ and in the 1995–1996 (84%)^27^ studies in which half and two-thirds of cases, respectively, were diagnosed among men younger than 40 years.

Leptospirosis on Seychelles is a severe disease leading to fatal outcomes mainly due to unusual and severe acute clinical manifestations such as pulmonary hemorrhage.^28,29^ However, the distribution of leptospirosis cases by age-group and by gender in Seychelles is very similar to that in Reunion Island. Another similarity is the high CFR in both islands (more than 11% in Seychelles, 3–5% in Reunion Island). Interestingly, the epidemiological situation on Seychelles and Reunion Island is quite distinct from that reported on Mayotte, an SWIO island part of the Comoros archipelago, in terms of gender ratio, age-group, and severity of the disease. Indeed, on Mayotte, female patients represent a third of cases^30,31^ and the CFR is significantly lower (0.9%). In Seychelles and Reunion Island, *L. interrogans* is responsible for the vast majority of human cases, whereas in Mayotte, *L. interrogans* is involved in a minority of severe cases.^32,33^ These contrasting features highlight the large differences that exist in the epidemiology of leptospirosis in the region, possibly resulting from the distinct virulence of *Leptospira* lineages/species prevailing on each island as recently substantiated through experimental infection.^34^

Besides, the leptospirosis CFR has decreased in Seychelles in the last decades (16% in 1992) similar to Reunion Island in the 1970s^16,26,35^ This decrease likely results from improvements in case management such as the development of ICUs and the generalization of modern resuscitation techniques such as dialysis and mechanical ventilation including extracorporeal membrane oxygenation.^35^ The CFR in Seychelles is still higher than that reported in Futuna (0.5%), Fiji, or Philippines (7%), similar to that reported in Mexico (12.8%) but lower than that in other areas of the world (19% in Taiwan).^30,31,36–40^ Pulmonary hemorrhage is considered as a predominant cause of death due to leptospirosis in Seychelles and Reunion Island. In our study, seven inpatients (13.5%) developed a pulmonary form, two of whom died, but the difference in the CFR was not statistically significant when compared with other clinical forms. Pulmonary forms, male gender, delayed treatment, thrombocytopenia, oliguria, and hemoptysis are associated with fatal cases in different studies worldwide.^38,39,41^ In our study, there was no difference in the delay before treatment between recovered and deceased leptospirosis cases. However, severe anemia and the presence of abdominal pain on admission were significantly associated with death. We also found that thrombocytopenia was more severe in fatal cases than recovering patients (an average platelet count of 49,000/μL versus 96,000/μL), but this difference was not statistically significant. Last, in our study, we were unable to find a higher mortality in severe forms when compared with uncomplicated forms, which is probably because of missing information for patients who died on admission.

Clinical (oliguria and jaundice) or biological (severe anemia, severe thrombocytopenia, high bilirubin, and renal failure) factors are associated with severe forms, as previously reported in different studies.^42–47^ We were not able to assess links with hypotension or coagulation abnormalities as this information was missing in the medical files. The delay in treatment is often associated with severe forms,^39,45,47^ although delays before treatment were very short in our study probably because of the small size of the country and access to free health facilities in most parts of the islands. Final clinical diagnosis was available only for 113 cases. The PPV of the clinical diagnosis of leptospirosis was low. It appears that more than 8% of leptospirosis cases were misdiagnosed, therefore without biological confirmation; the burden of leptospirosis would have been overestimated by 30%. Diagnosis was available for only 65 other causes of fever. More than 90% of those fevers were due to infectious diseases with respiratory infections, such as URTI, LRTI, and tonsillitis, accounting for more than 50% of other causes of fever. This explains why cough at the first examination was significantly associated with other causes of fever rather than leptospirosis.

Several domestic behaviors have been associated with leptospirosis risk worldwide. A recent meta-analysis has shown that footwear use decreases significantly the exposure to leptospirosis (OR = 0.59 [95% CI: 0.37–0.94]).^48^ Walking barefoot outside the house has been often associated with leptospirosis contamination worldwide,^49–52^ including in the SWIOIs.^30,32,53^ In our study, walking barefoot was not significantly linked to leptospirosis contrary to figures reported in a previous study in Seychelles.^53^ This is probably because of a decrease in this practice in the general population (22% in our sample, 26% in leptospirosis cases, and 20% in non-leptospirosis cases) compared with the precedent study (39% in leptospirosis cases and 17% in controls).

The presence of rats around houses or during outdoor activities is a known risk factor for leptospirosis in many temperate or tropical countries such as Hawaii and the SWIOIs.^30,31,54^ Although rats were predominantly found around houses of leptospirosis cases in 1993 (40% versus 25%) in Seychelles, the presence of rats reported during our study was not different around leptospirosis and non-leptospirosis cases. This situation suggests that rodents are not the main source of human contamination in Seychelles, as shown by a recent investigation.^16^ In our study, animals (variable including rats, cattle, poultry, cats, dogs, and rats) were present around 86% of leptospirosis cases compared with 65% around non-leptospirosis cases, but a significant difference was found only for poultry, cats, cattle, and dogs. Multivariate analysis of animal presence showed significant association of cattle (*P* < 0.0001) and cats (*P* < 0.05) around positive leptospirosis cases. Cattle are considered in many countries (Asia, east Africa, Indian subcontinent, Oceania, and Europe) as an important source of environmental contamination by *Leptospira*.^40,53–56^ Similarly, concerns about the role of pets in human leptospirosis are currently growing.^57^ In our study, cats were reported around all leptospirosis cases (OR = 96.9 [95% CI: 13.0–722.5]). Serological evidence of cat infection have been published in countries such as Chile,^58^ and cats can shed leptospires in their urine during acute clinical infection and may even act as chronic shedders.^59–61^ Furthermore, their involvement in the epidemiological cycle in rural areas is suspected.^62^ Dogs, mainly stray dogs, have been also suspected or are involved in the epidemiology of human leptospirosis worldwide, including in the SWIOIs.^33,49,57,63^ In 1995–1996, dogs were equally present around leptospirosis cases and controls, but in 2014–2015, dogs were found significantly more frequently (OR = 26.6 [95% CI: 3.6–198.3]) around leptospirosis cases (100%) than around non-leptospirosis cases (63%). Importantly, one dog in Seychelles was recently reported as a shedder of *L. interrogans*, genotype of which is found in most of the human cases but was noteworthily absent from hundreds of screened rats.^16^ Last, poultry were significantly (OR = 14.4 [95% CI: 6.6–31.3]) present more around leptospirosis cases than around non-leptospirosis cases. The presence of poultry or poultry breeding is often considered as due to exposure to rat droppings around poultry, as backyard poultry farming is a marker of the rural environment, which is in turn associated with leptospirosis contamination. Few studies are available about poultry leptospirosis, but a seroprevalence study conducted in Grenada and Trinidad showed that 11% of poultry harbored antibodies against *L.* spp., suggesting that chickens are exposed to leptospires.^64^ Furthermore, laboratory infections of chicken embryos showed that chicks that hatched from infected embryos developed a clinically recognizable leptospirosis and that leptospires can readily be observed in the circulating blood.^65^ Therefore, the possible role of cattle, poultry, cats, and dogs in human leptospirosis in Seychelles has to be investigated.

We did not find a significant difference in the proportion of subjects who had been working in the forest during the last 4 weeks before inclusion between leptospirosis cases and non-leptospirosis cases (35.6% versus 25.6%), contrary to the analyses conducted in the 1995–1996 study in Seychelles or in others part of the world.^31,32,66–68^ Gardening was reported as an activity at risk in Seychelles as in other parts of the world.^30–32,53,56^ The absence of link with professional gardening or gardening in the last 4 weeks might result from a lack of power of our study. Another explanation is that it may be easier to identify a link with a regular rather than with an accidental exposure. It is probably why we found a clear link between professions at high risk to leptospirosis that have a regular exposure and leptospirosis. Those people accounted for 61.7% of leptospirosis cases in our study and have about twice the odds of having leptospirosis (OR = 2.3 [95% CI: 1.2–4.5]). People practicing landscaping and/or farming as their main work activity accounted for about one-quarter of the total of leptospirosis cases, and more than half (53.8%) were confirmed leptospirosis cases. Compared with other main work activities, landscapers or farmers were at almost five times higher odds of having leptospirosis (OR = 5.4 [95% CI: 2.3–12.8]). This finding was also confirmed by multivariate analysis, where working as a landscaper or farmer was found to be significantly associated with leptospirosis cases (*P* < 0.0005).

In conclusion, our study confirms the heavy burden of the disease reported 20 years ago in Seychelles but highlights some striking differences in several epidemiological parameters that result at least in part from improvements in health care and in behavior changes. The development of health care has lowered case fatality of leptospirosis, despite a high disease incidence in the country. However, we report a rather low level of knowledge on leptospirosis, urging the need for implementing continuous information campaigns about this disease. Last, data suggest exposure to cattle, poultry, and pets as possible risk factors for the disease. As a recent study has shown that rats are probably not the main reservoir, complementary studies aiming at testing cattle and pets as additional reservoirs are paramount. These will allow for the development of a comprehensive picture of the overall human and animal epidemiology which is required to further refine preventive measures to mitigate the burden of this devastating disease.
